# Redefining cognitive testing: the impact of cognitive reserve and sex from early to late adulthood

**DOI:** 10.3389/fpsyg.2026.1735204

**Published:** 2026-03-06

**Authors:** Sonia Montemurro, Enrico Bovo, Giulia Sebastianutto, Giovanna Boccuzzo, Sara Mondini

**Affiliations:** 1 Department of Philosophy, Sociology, Education and Applied Psychology, University of Padua, Padua, Italy; 2 Department of Statistical Science, University of Padua, Padua, Italy; 3 IRCCS San Camillo Hospital, Venice, Italy

**Keywords:** aging, cognitive functioning, cognitive reserve, sex, testing

## Abstract

**Introduction:**

Cognition involves interconnected functions which may overlap across tasks. Thus, neuropsychological assessment should be optimized in tests, while integrating socio-demographic and socio-cultural factors like Sex and Cognitive Reserve (CR). This study aims to (1) determine whether a global cognitive factor can be identified from a comprehensive battery and whether it remains detectable after removing redundant tests; and (2) examine the combined effects of Age and Cognitive Reserve (CR) on performance across sexes.

**Methods:**

One thousand and one healthy individuals (599 females) aged 18–99 underwent a battery of tests and the Cognitive Reserve Index questionnaire (CRIq) was administered to estimate CR.

**Results:**

Reducing the number of overlapping tests revealed a single Cognition factor representing overall performance. Performance remained relatively stable up to the age of 60, then a marked decline was observed. Overall, test scores tended to be higher in males, and in most tasks, the higher CR the better the performance. However, when CR was considered, sex differences were no longer evident in most tasks. Notably, CR had a strong impact on female performance -especially CR gained through workrelated activities.

**Discussion:**

Reducing the number of redundant tests enhances the efficiency of the assessment. High CR reduces decline and slows its progression. Differences in sex-related performance seem to depend on CR, mainly in relation to occupation, which may differentially benefit males in terms of cognitive performance. This underscores the importance of promoting cognitively enriching life experiences for both sexes and equal career opportunities across the lifespan to support cognitive health in ageing.

## 1 Introduction

Global cognitive functioning encompasses multiple distinct components, such as attention, memory, language, and executive functions, which are interconnected and all together shape an individual’s cognitive profile.

Observable cognitive functioning, typically assessed through standardized tests, reflects the activity of multiple brain regions that are recruited in response to task demands (or, more broadly, to environmental demands). The way the brain interacts with observable cognitive manifestations has been extensively documented in the literature, both with regard to basic functioning and across a range of clinical conditions, as well as through different techniques for measuring neural signals (e.g., see the following recent systematic reviews and meta-analyses Marawi et al., 2023; Forkel et al., 2022; Jones et al., 2025). Cognitive functions do not arise from isolated brain regions but emerge from the continuous interplay among distributed and interconnected neural systems (Jonaitis et al., 2019). This perspective shifts the focus from static structures, which reduce mental processes to localized areas, to dynamic relationships. In fact, the theoretical need to clarify and distinguish different cognitive processes leads to a clear-cut distinction between psychometric tasks and their underlying cognitive dimensions, while obscuring the complex interactions among brain mechanisms. The different tasks typically included in standard neuropsychological batteries often exhibit a certain overlapping, assessing similar cognitive functions and leading to some redundancy. This suggests that a *comprehensive* cognitive assessment does not necessarily require a large number of tests, as the information gathered often overlaps across different measures. Furthermore, cognition is now considered one of the key aspects for evaluating the positive or negative outcomes of any intervention or treatment, including those primarily focused on physical health. For example, a cardiovascular intervention may be considered successful only when it is accompanied by the preservation of cognitive health (Gorodeski and Hashmi, 2020; Guo et al., 2025). However, premorbid cognitive conditions are in strong relation with a post-intervention status and thus the better the premorbid, the better the post-intervention expected conditions (e.g., Zangrossi et al., 2022). In these medical contexts, neuropsychology plays an essential role but cannot be overly extensive; rather, evaluations must be brief, highly focused, and accurate.

Such an effective evaluation should focus on optimizing or tailoring the process by selecting the most informative tasks in order to go straight to the core of cognitive functioning rather than merely accumulating data. For example, a test designed to measure working memory and executive functions might simultaneously tap into language comprehension and sustained attention abilities. These considerations raise the question of whether the dimensionality of full batteries may be reduced by identifying those subsets able to best capture inter-individual variability in cognitive functioning.

Only a few studies have investigated this issue. In Zangrossi et al. (2021) it has been shown for example that dimensionality reduction in clinical neuropsychological assessment can achieve high diagnostic accuracy. However, further research is still needed. An accurate diagnosis is particularly relevant in the context of ageing, where cognitive changes evolve across the lifespan and could be difficult to recognize and be given the right value in the complexity of the condition.

In addition to this, in early adulthood individuals generally achieve high cognitive performance, characterized by efficient processing speed, memory, and executive functioning (Hartshorne and Germine, 2015). However, with age, particularly in middle and later adulthood, some cognitive functions begin to decline (Salthouse, 2019), even if others can remain relatively preserved in their efficiency (Murman, 2015). Understanding cognitive decline in ageing requires considering the complex interplay of multiple aspects which influence an individual’s ability to adapt to and cope with such changes. Socio-demographic variables play a critical role in this context: they are informative predictors of cognitive capabilities and have gained increasing attention over the past two decades with regard to sex and education, but also to Cognitive Reserve (CR).

Based to the Collaboratory on Research Definitions for Reserve and Resilience in Cognitive Aging and Dementia, funded by the National Institute on Aging of the National Institutes of Health, CR reflects a property of the brain (e.g., molecular, cellular and network level) that allows for cognitive performance that is better than expected given the degree of life-course related brain changes (cognitively stimulating life-experiences and life-long learnings) and brain injury or disease: CR can be influenced by multiple genetic and environmental factors, operating at various points or continuously across the lifespan (Stern et al., 2023). In research, estimating what contributes to building CR takes into account knowledge, abilities and skills progressively acquired from experiences throughout the lifetime, whose counterpart is reflected in the richness and interconnectivity of the brain networks (i.e., brain reserve). Thus, higher CR may enhance resilience to cognitive decline by contributing to individual efficiency in cognitive performance (Gazes et al., 2023). This is why individuals the same age and sex may show different performances and why CR is an important factor to consider when investigating cognitive functioning in ageing (Stern, 2009; Stern et al., 2019).

CR is mainly derived by education, but also by occupation and leisure time activities carried out (Du et al., 2023; Oosterhuis et al., 2023). Notably, the quantification of life-experiences and practices, known to contribute to CR, can be approached through the use of self-reports and questionnaires, rather than through the observation of objective performance.

Cognitive reserve, as it is currently conceptualized in the literature, refers to resources potentially accumulated over time that are not solely attributable to formal schooling but extend across the entire lifespan. In the present study, education was not extracted from the more comprehensive CR questionnaire. In fact, although education represents an important component of cognitive reserve, it is considered here as a component of several other factors, which in this study contributed to a single global indicator (i.e., the CRI score).

In the literature, CR has been used for clinical purposes alongside sex to adjust raw scores based on normative samples (see Mondini et al., 2022a; Mondini et al., 2022b; Montemurro et al., 2023). However, it remains unclear how pervasive the influence of CR is across sexes.

Sex differences in cognition have been the subject of extensive research which has generally shown an advantage for males in most studies (see for example Giofrè et al., 2024; Reynolds et al., 2022). On the other hand, research has recently revealed both similarities and differences between the performance of males and females. For example, while general intelligence appeared to be comparable between sexes (Reynolds et al., 2022), some specific cognitive abilities have been shown to differ (Giofrè et al., 2024; Reynolds et al., 2022) tasks requiring inhibition and attention control (where women may outperform men) and spatial manipulation of stimuli (where men may outperform women).

Thus, a compelling question is to what extent persistent and long-standing disparities in education and employment opportunities have contributed to these differences, especially in contexts where women are still disadvantaged. Social and cultural influences such as gender stereotypes or educational opportunities might have affected CR and societal expectations. These may have encouraged males to engage more frequently in activities that develop spatial skills, while females may have received greater reinforcement in non-spatial fields (Jäncke, 2018; Valian, 1999). Recent research has emphasized the value of transdisciplinary approaches by integrating different fields to offer a more tailored understanding of the factors underlying sex differences and gender diversity in cognitive abilities (Kheloui et al., 2023). In fact, CR could shape how men and women have been differently exposed to and have benefited from the societal conditions that contribute to its accumulation. Using data from a large cohort of healthy adults, this study has a dual aim: 1. to determine whether the number of tests included in a comprehensive neuropsychological battery can be reduced to a selected informative subset of tests; 2. to investigate the combined influence of Age and CR on cognitive functioning across Sexes.

## 2 Methods

The whole sample is composed of 1,001 healthy individuals (599 females) aged between 18 and 99 (mean = 52,18 ± 20,25; Males: 18 to 95 years, mean = 48,95 ± 19.78; Females: 18 to 99 years, mean = 54,35 ± 20,29). They have been collected across different Italian regions between 2019 and 2021 (it should be noted that, in Italy, the restrictions associated with the COVID-19 pandemic were not continuous over time, and data collection was reasonably carried out during periods when conditions allowed); participants did not have any psychiatric or neurological disorders which could interfere with their cognitive functioning. The sample was tested with the complete Italian neuropsychological battery, the Brief neuropsychological examination (ENB-3; “Esame Neuropsicologico breve”; Mondini and Mapelli, 2022, Third edition) and with the Cognitive Reserve Index Questionnaire (Nucci et al., 2012). As reported in Mondini and Mapelli (2022), the battery, in its third version, has been validated on a wide population aged between 15 and 99 years and represents a reliable instrument for the present research.

The ENB-3 is an Italian neuropsychological battery composed of 16 sub-tests. The order of administration of each test is the following: 1. Digit span forward; 2. Digit span backward; 3. Trail making test A (TMT-A), 4. Copy drawing; 5. Interference memory (10 s), 6. Interference memory (30 s), 7. Abstract reasoning, 8. Verbal commands, 9. Immediate Prose memory, 10. Overlapping figures, 11. Praxis test, 12. Delayed Prose memory, 13. Spontaneous drawing, 14. Phonemic fluency, 15. Cognitive estimation, 16. Clock drawing.

Supplementary Section S1 includes more details about characteristics of the tests, with their minimum and maximum scores.

The *Cognitive Reserve Index questionnaire* (CRIq, Nucci et al., 2012) is a semi-structured interview that measures the potential reserve made up of resources gained during the lifespan. In a single index (i.e., CRI), the CRIq conveys the three primary sources of CR: education (CRI-*Education*), working activity (CRI-*WorkingActivity*), and leisure time activity (CRI-*LeisureTime*). The CRIq assigns a score to each item based on frequency and number of years of practice, adjusted for the variable Age through regression models. A full description of all the items included in the CRIq, the instructions for administration, and the scoresheets are available at https://www.cognitivereserveindex.org/NewEdition/.

The whole assessment session (ENB-3 and CRIq) of each participant lasted from 45 to 60 min depending on the resources and skills of the examinee. Data were collected by trained operators from the University of Padova (Italy). The study was approved by the Local Ethical Committee of the School of Psychology (University of Padua - Protocol N 3534) and it was conducted in accordance with the principles of the Declaration of Helsinki.

### 2.1 Statistical analyses

Descriptive analyses of the test scores were carried out. T-tests for the mean and Brown-Mood’s median tests (Hollander et al., 2013) were used to measure possible sex differences. Before carrying out a joint analysis of the 16 tests, we standardized them (using the formula $\frac{\left(T-\mu \right)}{\sigma }$ , where μ and σ refer to the mean and the standard deviation of the test, respectively).

The possibility to observe one or more cognitive patterns explained by the 16 tests was investigated by an Exploratory Factor Analysis (EFA). Following how CRI is calculated (on residuals regressed out of age, see Nucci et al., 2012), we removed the Age effect from the tests before analyzing them, thus using the residuals in linear models on the standardized tests that include Age as explanatory variable.

First, the factors resulting from the EFA were analyzed with respect to Age and CRI through linear regression models and separately for males and females. The linear regression model with factor F as dependent variable can be represented as:


$$F∼\text{Normal}(\mu ,\sigma^{2}),$$

$$\mu =\beta_{0}+\beta_{1}\mathrm{Age}+\beta_{2}\mathrm{CRI}+\beta_{3}\mathrm{Age}:\mathrm{CRI},$$

where $\beta_{0}$ , $\beta_{1}$ , $\beta_{2}$ , and $\beta_{3}$ are the four coefficients for the intercept, Age, CRI and their interaction, respectively.

In this way, we analyzed all the tests together from a general and comprehensive perspective, evaluating possible underlying structures. Subsequently, we proceeded to evaluate each test (in its original scale) individually, first observing any significant correlations with Age, CRI and Sex, and then using appropriate statistical models, that are Poisson regression models.

Given a single test score T, the Poisson model can be written as:


T∼Poisson(λ),

$$\log(\lambda )=\beta_{0}+\beta_{1}\mathrm{Age}+\beta_{2}\mathrm{CRI}+\beta_{3}\mathrm{Sex}+\text{interactions}$$

where $\beta_{0}$ , $\beta_{1}$ , $\beta_{2}$ , and $\beta_{3}$ are three regression coefficients related to the intercept and to the variables Age, CRI and Sex, while λ is the expected value of T . Quasi-Poisson regression model, rather than Poisson model (where the test scores show overdispersion) was used.” with “A Quasi-Poisson regression model, rather than Poisson model, was used when the test scores showed overdispersion. From the Poisson model it is possible to derive the rate ratios (RR) as exp(β) , i.e., the factor that measures the increase of the test-score with the unit increment of the explanatory variable. Statistical analyses were carried out using SAS (SAS Institute Inc., 2023) and R (R Core Team, 2021).

## 3 Results

### 3.1 Descriptive analyses

Scores at each single test (mean, standard deviation and range) relative to the whole sample and divided by Sex are reported in the Supplementary Sections S2, S3.

CRI of the whole sample ranged between 63 and 184 (mean = 107.90 ± 17.90; Males: mean = 109 ± 17.92; Females: mean = 107.18 ± 17.86).

No significant difference for Age and CRI-*Total score* were found across Sexes. Although CRI-*Education* did not differ across sexes, CRI-*WorkingActivity* was higher in Males than in females (mean *F* = 100; mean M = 107; *p* < 0.001) and CRI-*LeisureTime* was higher in Females (mean *F* = 113,2; mean *M* = 109,6; *p* < 0.01).

### 3.2 Factor analysis

Test scores of the 16 tests, standardized and adjusted by Age, showed an asymmetric distribution to the left: at least 50% of the population has values above the mean, with very low test scores for the remaining population.

The factor analysis revealed the presence of one main factor. Albeit two factors with an eigenvalue greater than 1 appeared, the first factor alone explained 38% of the variance, while the second explained only 7%. Moreover, the factorial pattern followed a parabolic trend, characteristic of the Guttman effect, which occurs when a two-factor solution is imposed despite the presence of one single factor explaining the variance. The appropriate checks for the assumptions of this analysis are explained in Supplementary Section S4.

A second factor analysis was performed after deleting 3 (out of the 16) tests that resulted highly correlated with other conceptually very similar tests (which evaluate similar cognitive constructs). This choice is also supported by the fact that very similar tests can exhibit collinearity. This second factor analysis was then carried out without the Digit Span forward, the Immediate Prose Memory, and the Interference Memory at 10 s. The Digit Span forward was indeed correlated with the Digit Span backward (*r* = 0.490); the Immediate Prose Memory with the Delayed Prose Memory (*r* = 0.767); the Interference Memory 10 s with the Interference Memory 30 s (*r* = 0.620).

The presence of one single important factor was confirmed in this second factor analysis, with one single eigenvalue greater than 1 explaining 37% of the total variance. Table 1 shows the factor pattern for the two analyses, including 16 and 13 tests, where each value represents the loading of each test on the main factor, i.e., how strongly each test is correlated with the main factor.

**Table 1:** Factor pattern showing the loadings of each cognitive test on the main factor, resulting from the two exploratory factor analyses (16 and 13 tests) using standardised tests with the effect of Age removed.

**Name**	**16 tests**	**13 tests**
Digit span forward	0.503	
Digit span backward	0.535	0.517
Trail making test A	0.659	0.678
Copy drawing	0.554	0.577
Interference memory (10 s)	0.716	
Interference memory (30 s)	0.701	0.682
Abstract reasoning	0.608	0.632
Verbal commands	0.66	0.671
Immediate prose memory (Immediate Recall)	0.715	
Overlapping figures	0.699	0.718
Praxis test	0.44	0.454
Delayed prose memory (Delayed Recall)	0.747	0.723.
Spontaneous drawing	0.362	0.385
Phonemic fluency	0.641	0.649
Cognitive estimation	0.529	0.544
Clock drawing	0.607	0.621

The factor analysis with 13 tests confirmed one single factor, which we named Cognition, indicating the level of cognitive performance or cognitive efficiency. This factor showed a similar distribution in Males and Females (see Supplementary Section S5). The Cognition factor showed a negative correlation with Age both in Males (*r* = −0.529, 95% CI –0.595, −0.54; *p* < 0.0001) and Females (*r* = −0.618, 95% CI –0.665, −0.565; *p* < 0.0001). The correlation between the Cognition factor and CR was positive in both Males (*r* = 0.104, 95% CI 0.006, 0.99; *p* = 0.0373) and Females (*r* = 0.319, 95% CI 0.245, 0.389; *p* < 0.0001).

We then proceeded to evaluate the effect of Age and CRI on the resulting factor, for both sexes, using a linear model. The checks of assumptions for linear regression and additional robustness analyses are reported in Supplementary Section S4. The results from the linear models showed that for persons aged 60 and over, as Age increased the Cognition factor decreased; the same was found in the younger individuals (<60 years old) but with a smaller effect.

**Figure 1:**
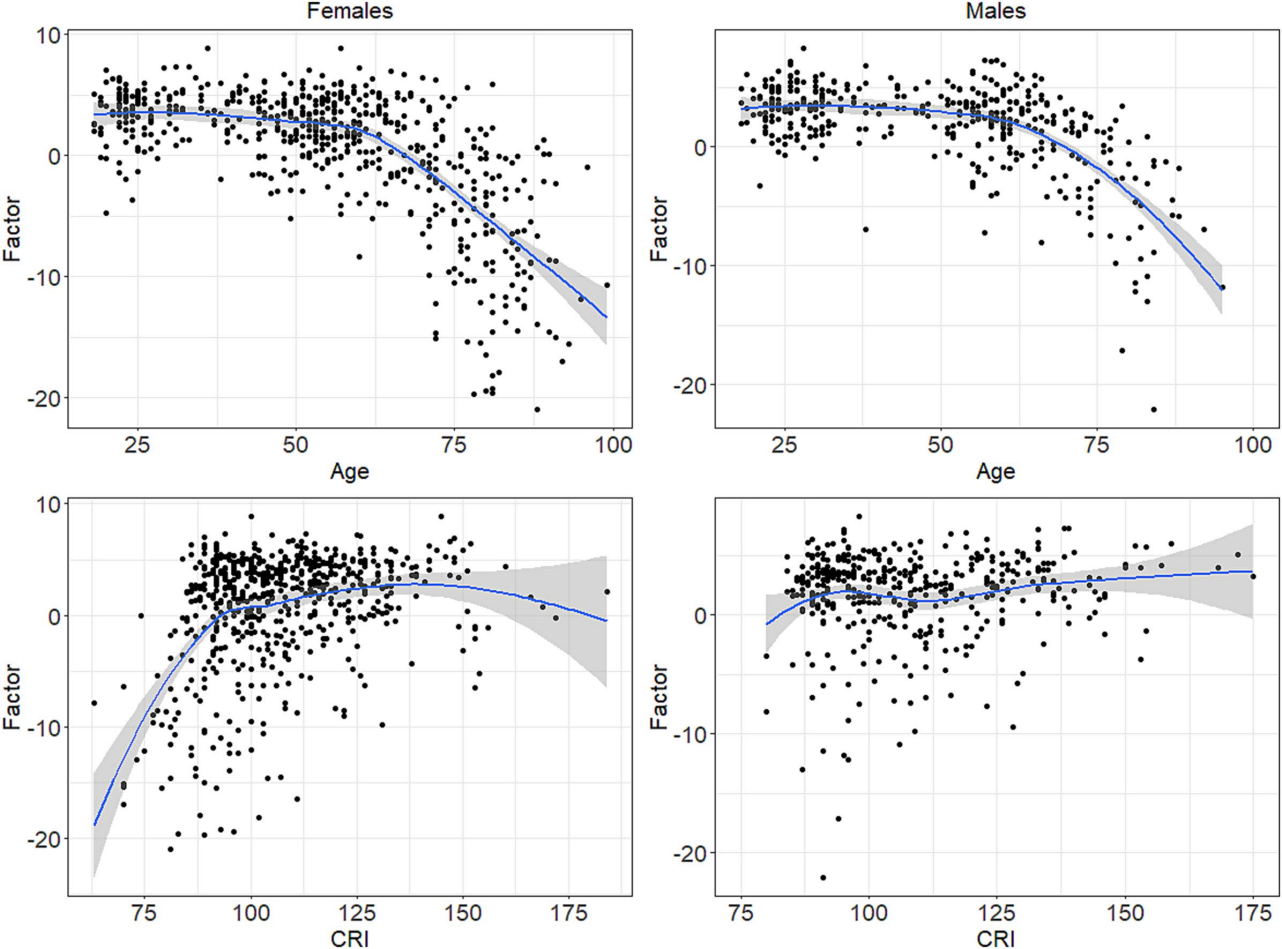
Relationship between Age and CRI with the Cognition Factor, relative to Females (left) and Males (right).

Furthermore, as CRI increased the Cognition factor increased, with effect for persons with lower CRI (i.e., CRI < 100) stronger than the same for persons with higher CRI (i.e., CRI ≥ 100); See Table 2. The prominent effect of Age in older persons and the stronger effect of CRI in persons with lower CR was shown in both males and females and no significant interaction between Age and CRI was observed. See Figure 1.

**Table 2:** Results of the linear model for the factor Cognition stratifying the population by Age higher or lower than 60 and by CRI higher or lower than 100, for Females and Males.

Population	β_0_ (Intercept)	β_1_ (Age)	β_2_ (CR)
Females			
Factor (≥60 y.o.)	5.564 (−0.519, 11.647)	−0.318 (−0.380, −0.255)	0.136 (0.110, 0.162)
Factor (<60 y.o.)	−1.885 (−3.523, −0.248)	−0.080 (−0.100, −0.060)	0.077 (0.059, 0.096)
Factor (CR ≥ 100)	0.904 (−1.688, 3.496)	−0.168 (−0.189, −0.147)	0.090 (0.067, 0.112)
Factor (CR < 100)	−10.883 (−18.168, −3.598)	−0.197 (−0.216, −0.177)	0.212 (0.135, 0.288)
Males			
Factor (≥60 y.o.)	10.309 (3.901, 16.716)	−0.323 (−0.387, −0.259)	0.101 (0.070, 0.132)
Factor (<60 y.o.)	−1.236 (−2.968, 0.496)	−0.093 (−0.118, −0.067)	0.074 (0.053, 0.096)
Factor (CR ≥ 100)	0.046 (−2.492, 2.583)	−0.137 (−0.159, −0.116)	0.081 (0.071, 0.092)
Factor (CR < 100)	−15.339 (−25.177, −5.500)	−0.187 (−0.211, −0.163)	0.257 (0.149, 0.364)

The estimates of the regression parameters and their 95% confidence intervals are shown.

### 3.3 Relation between age, CRI and sex in single test

After evaluating all the tests together from an overall perspective, we moved on to evaluating each test individually. First of all, we observed any significant correlations between each test and Age and CRI for both sexes.

Among the Females, results for all cognitive tests showed a negative correlation between Age and performance and a positive correlation between CRI and performance. Among the Males, a negative correlation between Age and performance was found, except in the Cognitive Estimation Test, while the correlation between performance and CR was generally non-significant (see Table 3).

**Table 3:** Correlations between the 13 tests and Age and CR, with associated *p*-values, reported separately for Females and Males.

Test	Females		Males	
	Correlation with age	Correlation with CR	Correlation with age	Correlation with CR
Digit span backward	−0.380 (*p* < 0.05)	0.147 (*p* < 0.05)	−0.358 (*p* < 0.05)	0.007
Trail making test A	−0.486 (*p* < 0.05)	0.116 (*p* < 0.05)	−0.447 (*p* < 0.05)	−0.063
Copy drawing	−0.397 (*p* < 0.05)	0.201 (*p* < 0.05)	−0.324 (*p* < 0.05)	0.016
Interference memory (30 s)	−0.522 (*p* < 0.05)	0.139 (*p* < 0.05)	−0.450 (*p* < 0.05)	−0.002
Abstract reasoning	−0.377 (*p* < 0.05)	0.254 (*p* < 0.05)	−0.268 (*p* < 0.05)	0.100 (*p* < 0.05)
Verbal commands	−0.448 (*p* < 0.05)	0.188 (*p* < 0.05)	−0.387 (*p* < 0.05)	0.081
Overlapping figures	−0.626 (*p* < 0.05)	0.178 (*p* < 0.05)	−0.572 (*p* < 0.05)	−0.028
Praxis test	−0.278 (*p* < 0.05)	0.105 (*p* < 0.05)	−0.240 (*p* < 0.05)	−0.015
Delayed prose memory (Delayed recall)	−0.555 (*p* < 0.05)	0.258 (*p* < 0.05)	−0.511 (*p* < 0.05)	−0.045
Spontaneous drawing	−0.215 (*p* < 0.05)	0.111 (*p* < 0.05)	−0.242 (*p* < 0.05)	0.069
Phonemic fluency	−0.452 (*p* < 0.05)	0.248 (*p* < 0.05)	−0.360 (*p* < 0.05)	0.080
Cognitive estimation	−0.192 (*p* < 0.05)	0.310 (*p* < 0.05)	−0.073	0.152 (*p* < 0.05)
Clock drawing	−0.512 (*p* < 0.05)	0.136 (*p* < 0.05)	−0.212 (*p* < 0.05)	0.041

Subsequently, we evaluated the effect of Age, Sex and CRI on each test through the use of appropriate statistical models. Poisson and quasi-Poisson models on the original test scores confirmed cognitive decline as Age increases (RR nearly always <1) and CR decreases (RR nearly always > 1), with no specific differences between Sexes (Table 4).

**Table 4:** Rate ratios (RR: increasing factor–or decreasing factor if less than 1–of the specific cognitive test when increasing one unit of Age and CRI, and when being a man compared to a woman) resulting from Poisson and quasi-Poisson models on the original scores of the 13 tests considering the effect of Age, Cognitive Reserve and Sex, with the corresponding 95% confidence intervals.

Test	RR (intercept)	RR (age)	RR (CR)	RR (male)
Entire sample				
Digit span backward	3.926 (3.292, 4.684)	0.992 (0.990, 0.994)	1.005 (1.003, 1.007)	
Trail making test A	186.441 (172.678, 201.301)	0.994 (0.993, 0.995)	1.003 (1.002, 1.004)	1.028 (1.002, 1.055)
Copy drawing	1.496 (1.130, 1.982)	0.994 (0.991, 0.996)	1.005 (1.002, 1.007)	
Interference memory (30 s)	6.037 (5.241, 6.955)	0.989 (0.988, 0.991)	1.006 (1.005, 1.008)	
Abstract reasoning	3.945 (3.343, 4.655)	0.994 (0.992, 0.995)	1.005 (1.004, 1.007)	
Verbal commands	4.361 (3.657, 5.200)	0.997 (0.995, 0.998)	1.002 (1.0005, 1.004)	
*Overlapping figures	24.164 (22.375, 26.096)	0.9894 (0.9887, 0.990)	1.006 (1.005, 1.007)	
Praxis test	5.873 (5.725, 6.025)			
Delayed prose memory (Delayed recall)	12.086 (11.058, 13.209)	0.990 (0.989, 0.991)	1.007 (1.006, 1.008)	0.950 (0.922,0.979)
Spontaneous drawing	1.919 (1.835, 2.007)			
*Phonemic fluency	10.899 (9.840, 12.072)	0.991 (0.990, 0.992)	1.007 (1.006, 1.008)	
Cognitive estimation	3.783 (3.177, 4.505)	1.003 (1.001, 1.005)	0.998 (0.996, 0.999)	
Clock drawing	7.854 (6.927, 8.905)	0.994 (0.992, 0.995)	1.004 (1.003, 1.006)	
60 years old and older				
Digit span backward	4.437 (2.501, 7.873)	0.990 (0.984, 0.996)	1.005 (1.003, 1.008)	
Trail making test A	447.981 (316.217,634.652)	0.983 (0.979, 0.986)	1.003 (1.001, 1.004)	
Copy drawing	2.1776 (0.8797, 5.3907)	0.988 (0.979, 0.997)	1.005 (1.001, 1.009)	
Interference memory (30 s)	13.038 (8.057, 21.098)	0.979 (0.974, 0.984)	1.006 (1.004, 1.008)	
Abstract reasoning	4.521 (3.088, 6.620)	0.990 (0.986, 0.994)	1.007 (1.005, 1.008)	
Verbal commands	7.121 (4.819, 10.521)	0.993 (0.988, 0.998)		
*Overlapping figures	46.418 (35.233 61.154)	0.982 (0.979, 0.985)	1.005 (1.004, 1.006)	
Praxis test	5.736 (5.501, 5.981)			
Delayed prose memory (Delayed recall)	18.309 (12.876, 26.034)	0.983 (0.980, 0.987)	1.007 (1.006, 1.009)	0.925 (0.867, 0.986)
Spontaneous drawing	1.856 (1.725, 1.998)			
*Phonemic fluency	21.300 (14.896, 30.458)	0.983 (0.979, 0.986)	1.007 (1.005, 1.008)	
Cognitive estimation	4.900 (2.861, 8.393)	0.994 (0.989, 0.999)	1.003 (1.001, 1.005)	
Clock drawing	2.1776 (0.8797, 5.3907)	0.988 (0.979, 0.997)	1.005 (1.001, 1.009)	1.127 (1.047, 1.214)
Younger than 60 years old				
Digit span backward	4.231 (3.284, 5.450)	0.993 (0.989, 0.996)	1.004 (1.001, 1.007)	
Trail making test A	221.170 (215.487, 227.0)	0.999 (0.9982, 0.9994)		
Copy drawing	1.900 (1.794, 2.012)			
Interference memory (30 s)	7.094 (5.804, 8.670)	0.995 (0.992, 0.998)	1.003	
Abstract reasoning	5.437 (5.256, 5.624)			
Verbal commands	4.790 (4.620, 4.965)			
*Overlapping figures	25.246 (22.832, 27.915)	0.992 (0.991, 0.994)	1.005 (1.004, 1.006)	
Praxis test	5.958 (5.769, 6.153)			
Delayed prose memory (Delayed recall)	14.224 (12.786, 15.822)	0.994 (0.993, 0.995)	1.004 (1.002, 1.005)	0.959 (0.930, 0.988)
Spontaneous drawing	1.958 (1.851, 2.071)			
*Phonemic fluency	11.560 (10.078, 13.261)	0.993 (0.992, 0.995)	1.006 (1.004, 1.007)	
Cognitive estimation	4.728 (4.560, 4.903)			
Clock drawing	9.621 (9.380, 9.869)			

RR obtained considering the entire sample and dividing the population into groups of persons aged 60 and over and persons younger than 60. The asterisk indicates that the quasi-Poisson model was adapted due to the overdispersion of the test scores.

Based on the above preliminary results showing a sharp decrease at 60 years old, regression analyses were then carried out dividing the participants between younger or older than 60. Results confirmed that in the older people (≥60 y.o.) Age had a significant negative effect on 11 out of 13 Tests while in the younger individuals (<60 y.o.) only in 6 out of 13 Tests. CRI had a significant positive effect in 10 Tests for the older people and only in 5 tests for the younger people. In addition, as shown in Table 4, Age and CRI showed stronger effects in the older than in the younger group. In the older group, a one-year increase led to a stronger decrease in scores, and one-point increase of CRI led to a higher test score. Also, Sex is significant for two tests in the older group (Delayed Prose Memory and Clock drawing) and for the Delayed Prose Memory in younger group. The Females scored better than the Males in the Delayed Prose Memory, while Males have better scores in Clock drawing. Interestingly, as shown in Table 4, no effect was found for Age and CRI in the Spontaneous Drawing and Praxis Tests, which remained stable with different levels of CRI. In the younger group, also the Verbal commands, Abstract reasoning, Cognitive Estimation, Copy of a Drawing and Clock Drawing tests are not affected by Age and CRI. The models were also evaluated considering the relationship among Sex, Age and CRI, but no interaction was found.

## 4 Discussion

This study had two main research aims. First, to verify whether a set of cognitive tests from a comprehensive neuropsychological battery can reflect a global cognitive factor and whether this factor remains identifiable after dimensionality reduction, i.e., by reducing the number of tests of neuropsychological assessment to obtain a more efficient and less redundant set of tests. Second, to investigate the combined influence of Age and CR on cognitive functioning across Sexes.

Results indicated the presence of a single global factor, interpretable as general cognitive functioning (i.e., Cognition factor) across both versions of the battery: when reducing the number of tests from 16 to 13, the factor structure remained unchanged, suggesting that the 13 retained measures were equally informative. In other words, reducing the number of variables may increase the likelihood of identifying a single underlying factor, reinforcing the decision to adopt the more parsimonious solution.

This result is consistent with previous studies in the literature. For example, studying a sample of 1,000 cognitively healthy individuals, Jonaitis et al. (2019) have shown significantly high correlations between test-scores assessing similar cognitive functions (e.g., Immediate and Delayed Memory). In line with earlier research (Donohue et al., 2014; Kozauer and Katz, 2013), they underline that keeping highly informative tests in comprehensive evaluations may not only reduce redundancy, but also enhance precision of measurements. Another study (Riello et al., 2021) outlined the relevance of integrating and critically interpreting the information derived from individual and global cognitive scores within the neuropsychological assessment. The authors highlighted that, rather than focus on the amount of administrable tests, it is crucial to make sharp, non-redundant evaluations when assessing cognitive functioning. Our data are also in line with some previous results shown by our research group (Zangrossi et al., 2021). Zangrossi and coauthors’ study, outlined heterogeneity of cognitive profiles in older adults with dementia by using a Factor Mixture Analysis (FMA). They showed how a smaller test-set of a whole neuropsychological battery may allow to critically distinguish sub-phenotypes of individuals diagnosed with dementia due to Alzheimer’s Disease from healthy older adults. Contrary to the common belief that an extensive pool of tests is necessary to reach an accurate diagnosis, previous results and present data on the healthy population may lead to redefine the amount of administered material and optimize the integration and interpretation of information (Dowling et al., 2010; Pessoa, 2022). It is also important to consider the history of each examinee (Riello et al., 2021) and also use an interpretative approach in the context of neuropsychology (Mondini et al., 2022a; Mondini et al., 2022b) which promotes a redefined focus based on agile integration of information.

The present research shows that from adulthood (18 y.o. and over), cognitive performance remains relatively stable or shows a minor decline, which becomes pronounced after the age of 60, when the age effect is up to four times greater than that observed in younger individuals. A positive correlation is shown between cognitive performance and CR, with higher cognitive performance related to higher CRI score. Even a little amount of CR seems to foster cognitive performance.

Overall, these results are in line with previous research. The detrimental effect of Age and the potentially compensatory mechanisms driven by CR have been found in vulnerable individuals, i.e., in older adults with low CR whose frailty is due to age (Ansado et al., 2013). In this context, Ansado et al. (2013) have previously highlighted how the cognitive system of healthy older adults may naturally adopt compensatory mechanisms; however, when such processes are insufficient to cope with increased demands or environmental challenges, they may draw on their CR.

In our research, regression analyses were performed to assess the role of Age and CR together with Sex, by using Cognitive performance as a dependent variable in different tests. While Sex differences were visible in individual cognitive test performance (Males outperformed Females), such differences almost disappeared when CR was considered, revealing how differences in CR rather than mere differences across sexes had a direct impact on cognitive performance. The effect of sex remains significant only in the Delayed Prose Memory test in both age groups, where females score better than males, and in the Clock Drawing test in the older group, where males perform better than females.

Overall, the finding of this research underlines how cognitive functioning in ageing can be considered the result of a complex interplay among socio-demographic features, an interplay that cannot be reduced to basic differences between sexes. Cognitive functioning reflects one’s own social background, which is inevitably linked to access and exposure to cognitively stimulating life experiences.

Although recent studies still report significant differences in cognition between males and females (Giofrè et al., 2024; Reynolds et al., 2022) when it comes to aging, it is essential to consider the resources accumulated throughout life and available to the individual as they serve as significant modulators of cognitive efficiency in older adults.

## 5 Conclusion

This study, based on a large sample of 1,001 Italian individuals aged 18–99, shows that: (1) The global information derived from an extensive neuropsychological battery can be optimized and reduced to a smaller set of tests, while still capturing a single global cognitive factor as effectively as a larger battery of tests; (2) Cognitive performance remains relatively stable until the age of 60, at which age a sharp decline is observed; (3) Age and Cognitive Reserve do influence cognitive functioning, particularly in the most vulnerable portions of the ageing population, that is, older adults with lower CR; (4) although spurious comparison between males and females suggest that males outperform females in most cognitive tasks, when CR is taken into account such differences almost dissipate in both older and younger individuals, except for two tests, where performance is higher for males in one and for females in the other.

This difference is likely due to women’s comparatively lower access to occupational opportunities, as confirmed by the results of this study, which is reflected by the component of CR related to working activities in the female participants. Overall, this study advocates for a wider integrated and critical approach to neuropsychological assessment that consistently takes into account the complex interaction among socio-demographic variables that influence ageing. Such an approach would help to support tailored and person-centered practices in both research and clinical settings.

## Data Availability

The raw data supporting the conclusions of this article will be made available by the authors under request, without undue reservation.

## References

[ref1] Ansado J. Monchi O. Ennabil N. Deslauriers J. Jubault T. Faure S. . (2013). Coping with task demand in aging using neural compensation and neural reserve triggers primarily intra-hemispheric-based neurofunctional reorganization. Neurosci. Res. 75, 295–304. doi: 10.1016/j.neures.2013.01.012, 23453977

[ref2] Donohue M. C. Sperling R. A. Salmon D. P. Rentz D. M. Raman R. Thomas R. G. . (2014). The preclinical Alzheimer cognitive composite: measuring amyloid-related decline. JAMA Neurol. 71, 961–970. doi: 10.1001/jamaneurol.2014.803, 24886908 PMC4439182

[ref3] Dowling N. M. Hermann B. La Rue A. Sager M. A. (2010). Latent structure and factorial invariance of a neuropsychological test battery for the study of preclinical Alzheimer’s disease. Neuropsychology 24, 742–756. doi: 10.1037/a0020176, 21038965 PMC3057903

[ref4] Du C. Miyazaki Y. Dong X. Li M. (2023). Education, social engagement, and cognitive function: a cross-lagged panel analysis. J. Gerontol. 78, 1756–1764. doi: 10.1093/geronb/gbad088, 37294899 PMC10561888

[ref5] Forkel S. J. Friedrich P. Thiebaut de Schotten M. Howells H. (2022). White matter variability, cognition, and disorders: a systematic review. Brain Struct. Funct. 227, 529–544. doi: 10.1007/s00429-021-02382-w, 34731328 PMC8844174

[ref6] Gazes Y. Lee S. Fang Z. Mensing A. Noofoory D. Hidalgo Nazario G. . (2023). Effects of brain maintenance and cognitive reserve on age-related decline in three cognitive abilities. J. Gerontol. 78, 1284–1293. doi: 10.1093/geronb/gbad044, 36882044 PMC10394982

[ref7] Giofrè D. Toffalini E. Esposito L. Cornoldi C. (2024). Sex/gender differences in general cognitive abilities: an investigation using the Leiter-3. Cogn. Process. 25, 663–672. doi: 10.1007/s10339-024-01199-9, 38748044 PMC11541283

[ref8] Gorodeski E. Z. Hashmi A. Z. (2020). Integrating assessment of cognitive status in elderly cardiovascular care. Clin. Cardiol. 43, 179–186. doi: 10.1002/clc.23318, 31845363 PMC7021647

[ref9] Guo J. Zhang Z. Meng X. Jing J. Hu Y. Yao Y. . (2025). Atrial fibrillation catheter ablation, brain glymphatic function, and cognitive performance. Eur. Heart J. 46, 1733–1743. doi: 10.1093/eurheartj/ehaf036, 39981927

[ref10] Hartshorne J. K. Germine L. T. (2015). When does cognitive functioning peak? The asynchronous rise and fall of different cognitive abilities across the life span. Psychol. Sci. 26, 433–443. doi: 10.1177/0956797614567339, 25770099 PMC4441622

[ref11] Hollander M. Wolfe D. A. Chicken E. (2013). Nonparametric statistical methods. Hoboken, New Jersey, USA: John Wiley & Sons.

[ref12] Jäncke L. (2018). Sex/gender differences in cognition, neurophysiology, and neuroanatomy. F1000Res 7:805. doi: 10.12688/f1000research.13917.1, 29983911 PMC6013760

[ref13] Jonaitis E. M. Koscik R. L. Clark L. R. Ma Y. Betthauser T. J. Berman S. E. . (2019). Measuring longitudinal cognition: individual tests versus composites. Alzheimers Dementia 11, 74–84. doi: 10.1016/j.dadm.2018.11.006, 31673596 PMC6816509

[ref14] Jones B. D. Gallucci J. Jones O. Y. Zhukovsky P. Wong S. Lakhani K. . (2025). Associations between structural brain measures and cognitive function in bipolar disorder: a systematic review and meta-analysis. Neuropsychopharmacology 50, 1256–1264. doi: 10.1038/s41386-025-02096-1, 40274973 PMC12170891

[ref15] Kheloui S. Jacmin-Park S. Larocque O. Kerr P. Rossi M. Cartier L. . (2023). Sex/gender differences in cognitive abilities. Neurosci. Biobehav. Rev. 152:105333. doi: 10.1016/j.neubiorev.2023.105333, 37517542

[ref16] Kozauer N. Katz R. (2013). Regulatory innovation and drug development for early-stage Alzheimer’s disease. N. Engl. J. Med. 368, 1169–1171. doi: 10.1056/NEJMp1302513, 23484795

[ref17] Marawi T. Ainsworth N. J. Zhukovsky P. Rashidi-Ranjbar N. Rajji T. K. Tartaglia M. C. . (2023). Brain-cognition relationships in late-life depression: a systematic review of structural magnetic resonance imaging studies. Transl. Psychiatry 13:284. doi: 10.1038/s41398-023-02584-2, 37598228 PMC10439902

[ref18] Mondini S. Cappelletti M. Arcara G. (2022a). Methodology in neuropsychological assessment. London, UK: Taylor & Francis Ltd.

[ref19] Mondini Sara Mapelli Daniela. (2022). Esame Neuropsicologico Breve 3—Autori-vari—Raffaello Cortina Editore—Libro Raffaello Cortina Editore. Available online at: https://www.raffaellocortina.it/scheda-libro/autori-vari/esame-neuropsicologico-breve-3-9788832854831-3806.html

[ref20] Mondini S. Montemurro S. Pucci V. Ravelli A. Signorini M. Arcara G. (2022b). Global examination of mental state: an open tool for the brief evaluation of cognition. Brain Behav. 12:e2710. doi: 10.1002/brb3.2710, 35861637 PMC9392517

[ref21] Montemurro S. Mondini S. Pucci V. Durante G. Riccardi A. Maffezzini S. . (2023). Tele-global examination of mental state (tele-GEMS): an open tool for the remote neuropsychological screening. Neurol. Sci. 44, 3499–3508. doi: 10.1007/s10072-023-06862-1, 37248426 PMC10226870

[ref22] Murman D. L. (2015). The impact of age on cognition. Semin. Hear. 36, 111–121. doi: 10.1055/s-0035-1555115, 27516712 PMC4906299

[ref23] Nucci M. Mapelli D. Mondini S. (2012). Cognitive reserve index questionnaire (CRIq): a new instrument for measuring cognitive reserve. Aging Clin. Exp. Res. 24, 218–226. doi: 10.3275/7800, 21691143

[ref24] Oosterhuis E. J. Slade K. May P. J. C. Nuttall H. E. (2023). Toward an understanding of healthy cognitive aging: the importance of lifestyle in cognitive reserve and the scaffolding theory of aging and cognition. J. Gerontol. Ser. B. 78, 777–788. doi: 10.1093/geronb/gbac197, 36546399 PMC10174283

[ref25] Pessoa L. (2022). The entangled brain: How perception, cognition, and emotion are woven together. Cambridge, Massachusetts, USA: The MIT Press.

[ref26] R Core Team (2021). R: A language and environment for statistical computing. R foundation for statistical computing, Vienna, Austria. Available online at: https://www.R-project.org/ (Accessed October 1, 2025).

[ref27] Reynolds M. R. Hajovsky D. B. Caemmerer J. M. (2022). The sexes do not differ in general intelligence, but they do in some specifics. Intelligence 92:101651. doi: 10.1016/j.intell.2022.101651

[ref28] Riello M. Rusconi E. Treccani B. (2021). The role of brief global cognitive tests and neuropsychological expertise in the detection and differential diagnosis of dementia. Front. Aging Neurosci. 13:648310. doi: 10.3389/fnagi.2021.648310, 34177551 PMC8222681

[ref29] Salthouse T. A. (2019). Trajectories of normal cognitive aging. Psychol. Aging 34, 17–24. doi: 10.1037/pag0000288, 30211596 PMC6367038

[ref30] SAS Institute Inc. (2023). SAS/STAT® 15.3 User’s Guide. Cary, NC: SAS Institute Inc.

[ref31] Stern Y. (2009). Cognitive reserve. Neuropsychologia 47, 2015–2028. doi: 10.1016/j.neuropsychologia.2009.03.004, 19467352 PMC2739591

[ref32] Stern Y. Albert M. Barnes C. A. Cabeza R. Pascual-Leone A. Rapp P. R. (2023). A framework for concepts of reserve and resilience in aging. Neurobiol. Aging 124, 100–103. doi: 10.1016/j.neurobiolaging.2022.10.015, 36653245 PMC10424718

[ref33] Stern Y. Barnes C. A. Grady C. Jones R. N. Raz N. (2019). Brain reserve, cognitive reserve, compensation, and maintenance: operationalization, validity, and mechanisms of cognitive resilience. Neurobiol. Aging 83, 124–129. doi: 10.1016/j.neurobiolaging.2019.03.022, 31732015 PMC6859943

[ref34] Valian V. (1999). Why so slow?: The advancement of women. Cambridge, Massachusetts, USA: MIT Press.

[ref35] Zangrossi A. Montemurro S. Altoè G. Mondini S. (2021). Heterogeneity and factorial structure in Alzheimer’s disease: a cognitive perspective. J. Alzheimers Disease 83, 1341–1351. doi: 10.3233/JAD-210719, 34420975

[ref36] Zangrossi A. Silvestri E. Bisio M. Bertoldo A. De Pellegrin S. Vallesi A. . (2022). Presurgical predictors of early cognitive outcome after brain tumor resection in glioma patients. NeuroImage 36:103219. doi: 10.1016/j.nicl.2022.103219, 36209618 PMC9668620

