# Supplementary material for: Redefining cognitive testing: the impact of cognitive reserve and sex from early to late adulthood
Source: Front Psychol. 2026 Mar 6;17:1735204. doi: 10.3389/fpsyg.2026.1735204 (PMC13003597; doi:10.3389/fpsyg.2026.1735204)
Supplement: Supplementary file 1 [file Table_1.pdf]

SUPPLEMENTARY MATERIALS

Redefining cognitive Testing: the impact of cognitive reserve and sex from early to late adulthood

Sonia Montemurro[1], Enrico Bovo[2], Giulia Sebastianutto[1], Giovanna Boccuzzo[2], Sara Mondini[1,3]

1 Department of Philosophy, Sociology, Education and Applied Psychology, University of Padua, Italy

2 Department of Statistical Science, University of Padua, Italy

3 IRCCS San Camillo Hospital, Venice, Italy

SECTION 1

S1.1 Description of the tests of ENB-3

The ENB-3 (Esame Neuropsicologico Breve-3) is a standardized neuropsychological battery used for the assessment of different cognitive domains. Each subtest mainly targets a cognitive function, with the administration time ranging from approximately 45 - 60 minutes. A summary of the tests is shown below:

Name of the test	Main Cognitive Functions involved	Task	Duration
**Digit Span Forward**	Verbal short-term and working memory	Repetition of sequences of digits in ascending order	3–5 min
**Digit Span Backward**	Verbal short-term and working memory	Repetition of sequences of digits in reverse order	3–5 min
**Trail Making Test A**	Attention, processing speed, cognitive flexibility	Connecting numbers in ascending order as fast as possible	5–7 min
**Copy Drawing**	Visuoconstructional skills	Copying the figure of a house with some details to include	5 min (included)
**Interference Memory (10 seconds)**	Inhibitory control and selective attention	Repeating triplets of letters and recalling them after counting by twos for 10 seconds	2–3 min
**Interference Memory (30 seconds)**	Inhibitory control and selective attention	Repeating triplets of letters and recalling them after counting by twos for 30 seconds	2–3 min
**Abstract Reasoning**	Abstract reasoning and conceptualization	Identifying the underlying relationship among different concepts	3–5 min

Name of the test	Main Cognitive Functions involved	Task	Duration
**Verbal Commands**	Auditory comprehension	Following increasingly complex verbal instructions using tokens	3–5 min
**Immediate Prose Memory (Immediate Recall)**	Verbal episodic memory	Recalling the information of a story immediately	6 min
**Overlapping Figures**	Visual discrimination and perceptual organization	Identifying individual figures overlapping	3–5 min
**Praxis Test**	Motor planning and execution	Performing gestures	3–5 min
**Delayed Prose Memory (Delayed Recall)**	Verbal episodic memory	Recalling the information of a story after a delay	6 min
**Spontaneous Drawing**	Visuoconstructional and planning abilities	Drawing a figure	2–3 min

Name of the test	Main Cognitive Functions involved	Task	Duration
**Phonemic Fluency**	Lexical access and executive function	Producing as many words as possible under phonemic constraints within 1 minute	3–5 min
**Cognitive Estimation**	Executive function and reasoning	Providing estimates for questions where exact answers are unknown (e.g., length of a guitar)	3–5 min
**Clock Drawing**	Visuoconstructional skills	Drawing a clock	5 min (included)

S1.2 Minimum and Maximum Scores for ENB-3 Neuropsychological Tests

The following table presents the minimum and maximum achievable scores for the 16 tests included in the ENB-3 battery.

Name	Minimum Score	Maximum Score
**Digit Span Forward**	0	8
**Digit Span Backward**	0	8
**Trail Making Test A**	999 sec if not executable	No fixed maximum (execution time measured)
**Copy Drawing**	0	2
**Interference Memory (10 seconds)**	0	9
**Interference Memory (30 seconds)**	0	9
**Abstract Reasoning**	0	6
**Verbal Commands**	0	5
**Immediate Prose Memory (Immediate Recall)**	0	21
**Overlapping Figures**	0	50

Name	Minimum Score	Maximum Score
**Praxis Test**	0	6
**Delayed Prose Memory (Delayed Recall)**	0	21
**Spontaneous Drawing**	0	2
**Phonemic Fluency**	0	-
**Cognitive Estimation**	0	5
**Clock Drawing**	0	10

S2. SECTION

The table shows the descriptive statistics of the 16 tests.

Name of the test	Minimum	Mean	Median	Maximum	Standard Deviation
Digit span forward	0	5,96	6	8	1,11
Digit span backward	0	4,53	4	8	1,48
Trail making test A (TMT-A)	0	197	210	232	43,52
Copy drawing	0	1,78	2	2	0,46

Interference memory (10 secs)	0	7,64	9	9	2,03
Interference memory (30 secs)	0	7,02	8	9	2,34
Abstract reasoning	0	5,11	6	6	1,27
Verbal commands	0,5	4,68	5	5	0,55
Immediate prose memory	1	12,35	13	21	4,08
Overlapping figures	1	27,59	28	50	7,58
Praxis test	2	5,87	6	6	0,41
Delayed prose memory	0	14,62	16	21	4,24
Spontaneous drawing	0	1,92	2	2	0,29
Phonemic fluency	0	14,87	15	30	4,78
Cognitive estimation	1	4,64	5	5	0,67
Clock drawing	0	8,9	10	10	2,14

S3. SECTION. Descriptive analyses of the cognitive variables and sex-difference.

Test	Sex	Minimum	Mean	Maximum	Standard deviation	Differences (t test)
**Digit Span Forward**	Female	0	5.80	8	1.10	M > F p<0.001
	Male	3	6.19	8	1.09	
**Digit Span Backward**	Female	0	4.37	8	1.45	M > F p<0.001
	Male	0	4.76	8	1.49	
**Trail Making Test – A (TMT-A)**	Female	0	191.80	232	48.86	M > F p<0.001
	Male	0	204.80	232	32.59	
**Copy Drawing Test**	Female	0	1.74	2	0.49	M > F p<0.001
	Male	0	1.85	2	0.39	
**Memory Interference Test (10 sec)**	Female	0	7.40	9	2.18	M > F p<0.001
	Male	0	8	9	1.72	
**Memory Interference Test (30 sec)**	Female	0	6.72	9	2.47	M > F p<0.001
	Male	0	7.47	9	2.05	
**Abstraction Test**	Female	0	5.05	6	1.39	No differences p=0.065
	Male	0	5.19	6	1.07	
**Verbal Commands Test**	Female	2	4.64	5	0.57	M > F p=0.002
	Male	0.5	4.75	5	0.53	
**Overlapping Figures**	Female	4	27	50	7.86	M > F p=0.002
	Male	1	28.46	46	7.07	

**Praxis Test**	Female	2	5.85	6	0.47	M > F<br>p=0.029
	Male	4	5.91	6	0.32	
**Delayed Prose Memory**	Female	0	14.57	21	4.40	No differences<br>p=0.632
	Male	2	14.7	21	4.01	
**Spontaneous Drawing**	Female	0	1.92	2	0.28	No differences<br>p=0.695
	Male	0	1.91	2	0.30	
**Phonemic Fluency**	Female	0	14.63	30	4.92	M > F<br>p=0.044
	Male	0	15.24	29	4.54	
**Cognitive Estimation**	Female	1	4.55	5	0.75	M > F<br>p<0.001
**Clock Drawing**	Female	0	8.65	10	2.44	M > F<br>p<0.001
	Male	0	9.26	10	1.52	

SECTION S4

To perform the Exploratory Factor Analysis, certain characteristics were examined to verify compliance with the assumptions of the analysis: sampling adequacy, multivariate normality, correlations among tests, multicollinearity, linearity, and communality.

The sample is adequate thanks to an N equal to 1001, which is equivalent to approximately 62 observations per variable in the case of the 16 tests. Multivariate normality, on the other hand, was not checked, as the EFA performed was based on PCA.

The correlations among the tests are sufficiently large, with a correlation matrix that is clearly different from the identity matrix, but they never exceed 0.76, thus ruling out possible multicollinearity.

The assumption of linearity requires a linear relationship between each test and the factor, which, being latent, is not directly observable; therefore, linearity among the various tests is evaluated empirically. In this context, the items were standardized; however, as is often the case in psychometric practice, the items are discrete in nature, and the relationship between item and factor is modeled using correlations. Thanks to the robustness of EFA, especially in large samples, and the empirical support of the results leading to a single interpretable factor, any deviations from linearity are not such as to invalidate the analysis.

Finally, the communalities range between 0.2 and 0.5, indicating that the single factor explains a good proportion of the variance in the observed variables.

To correctly use the linear regression model to assess the effect of age and CRI on the Cognition Factor, certain assumptions must be verified. Specifically, these assumptions are linearity, homoscedasticity, outliers, and normality of residuals.

At this stage of the analysis, eight models were fitted, since both sexes were considered and thresholds of 60 for Age and 100 for CRI were set, and the assumptions were then verified for each of them.

The assumption of linearity was evaluated by observing the pattern of the model residuals with respect to the predicted values and with respect to the predictors Age and CRI, and it was always met.

Similarly, homoscedasticity, that is, the constancy of the variance of the residuals, was verified. In all cases, the behaviour of the residuals showed no substantial differences in terms of variability.

The presence of outliers was verified by studying the standardised residuals, which showed the presence of a few outliers; however, after evaluating leverage and Cook's distance, an appropriate sensitivity analysis showed that the outliers detected did not change the estimates of the model parameters.

Given that the individual tests contributing to the Cognition Factor exhibited asymmetric distributions, the robustness of the regression models was explicitly assessed. It should be noted that the analyses focus on the Cognition factor itself, which, as a weighted linear combination derived from an exploratory factor analysis, exhibited a more regular and approximately symmetric distribution compared to the individual tests. In addition, the regression models were fitted on subsamples defined by age (above and below 60 years) and CRI (above and below 100), resulting in less asymmetric distributions.

Furthermore, to evaluate residual asymmetry, heteroskedasticity, or influential observations effects, each model was re-estimated using heteroskedasticity-consistent (HC3) robust standard errors, which are specifically designed to account for potential heteroskedasticity and leverage. The results were virtually identical to those obtained under classical ordinary least squares inference, indicating robustness to skewness in the individual tests.

Finally, the normality of the residuals was assessed using normal Q-Q plots and the Shapiro-Wilk test. In all cases, the Q-Q plots did not show substantial deviations from normality, whereas the Shapiro-Wilk test was significant due to the large sample size. We therefore considered that any deviations from normality were limited and did not affect the results.

S5 SECTION

Factor score resulting from the two factor analyses on the 13 standardized tests with the effect of age removed for females and males. The table shows the main statistics for the factor resulting from the factor analysis using the entire sample and distinguishing by sex.

	**Total**	**Females**	**Males**
Minimum	-23.01	-21.00	-22.10
Mean	1.10	0.53	1.81
Median	2.52	2.15	2.78
Maximum	8.80	8.87	8.29
Standard deviation	4.78	5.35	3.78
